# Supplementary material for: The impact of whole genome duplications on the human gene regulatory networks
Source: PLoS Comput Biol. 2021 Dec 6;17(12):e1009638. doi: 10.1371/journal.pcbi.1009638 (PMC8675932; doi:10.1371/journal.pcbi.1009638)
Supplement: Attachment — Submitted filename: Mottes_PLoS_Responses_to_the_reviewers.pdf [file pcbi.1009638.s002.pdf]

To the kind attention of

Dr. Yamir Moreno
Associate Editor
PLoS Computational Biology

Dr. Jason Papin
Editor-in-Chief
PLoS Computational Biology

PCOMPBIOL-D-21-01527
The impact of whole genome duplications on the human gene regulatory networks

Dear Mr Mottes,

Thank you for submitting your manuscript "The impact of whole genome duplications on the human gene regulatory networks" for consideration at PLOS Computational Biology. As with all papers reviewed by the journal, your manuscript was reviewed by members of the editorial board and by several independent reviewers. The reviewers appreciated the attention to an important topic. Based on the reviews, we are likely to accept this manuscript for publication, providing that you modify the manuscript according to the review recommendations.
Please prepare and submit your revised manuscript within 30 days.
[. . .]

Sincerely,

Yamir Moreno
Associate Editor
PLOS Computational Biology

Jason Papin
Editor-in-Chief
PLOS Computational Biology

Dear Dr. Moreno and Dr. Papin,

We are very pleased of the positive comments of the Reviewers and grateful for their work on our manuscript. We have carefully considered their suggestions and made revisions to our work accordingly. The changes to the manuscript have been highlighted in red in the revised version, while below this letter you can find our point-by-point response.

Yours sincerely,

Torino, November 2, 2021
Francesco Mottes* (on behalf of all authors)

* Physics Department, INFN and University of Torino
Via P. Giuria 1.
10125 Torino, Italy
e-mail: francesco.mottes@gmail.com

TO THE REVIEWERS

We thank the two Reviewers for their positive and constructive comments regarding our work. We revised the paper following their suggestions. We are convinced that their remarks were useful, and resulted in an improved version of our work that better convey the main messages.

ANSWERS TO REVIEWER #1

Using human paralogues, this work compares those originating from 2 ancestral Whole Genome Duplications (WGD), which predate the origin of vertebrates, with those originating from Small Scale duplications of similar age. The analysis uses 3 kinds of networks (transcription regulatory networks, protein-protein interaction networks, and miRNA interaction networks). Common units of interaction or regulation are extracted as "network motifs". The main result is that some classes of motifs are differentially enriched, indicating that the two types of duplications play different roles in the evolution of regulation. The authors further discuss how the different motifs can arise, lead to different types of regulation, and can interact to create more complex regulatory structures. The analysis is similar to a previous one that studied another WGD in yeast. However, this is the first one to study WGD in vertebrates and has significant consequences to our understanding of regulatory networks in humans. In this manuscript, the problem is clearly stated, due credit is given to previous work, and the discussion of the subject is enriched with new insights.
The datasets seem well thought and clean enough. The authors were careful enough to only include duplications of comparable age. One of their first results was that the connectedness (the node degree) was similar for all the datasets compared. This is important because the statistic could be affected by the degree of the nodes. Controls, statistical methods, and null models are correct. In most results obtained, the Z-scores indicate extremely high significance.

We are grateful to the Reviewer for the positive remarks.

In my opinion, the work is solid and sufficient. I would have liked to see some discussion of specific genes given as examples. That would make the implications of the study more tangible to many readers.

We thank the Reviewer for the kind suggestion, we agree that the addition of an illustrative example could facilitate the understanding of our work. We therefore added in the revised manuscript a detailed discussion of the RAR/RXR pathway, which we consider a splendid example of the beneficial effects that WGD events introduced in the vertebrate regulatory networks. To better ground our discussion of this example we also added some references, reported below as [1–5]. The choice of this particular pathway was also driven by the extremely high statistical significance of the overlap

of its gene regulatory interactions. A hypergeometric test conducted on the common protein-protein interactions between any two genes in the RAR family gives a p-value of the order of 10^{-35} or less.

The authors have set a GitHub repository to provide access to their data. One dataset that is missing there, and also not adequately described in Materials and Methods is that of the non-duplicated genes.

We thank the reviewer for pointing this out. We added in the revised manuscript a brief section in Materials and Methods, describing how we construct non-duplicated gene couples. We essentially consider as non-duplicated couples all of the couples that can be constructed with the genes of a network that are neither SSD nor WGD couples. For this reason, the number of couples that were generated for the analysis was of the order of at least 10^7. We decided not to upload these very large files of not-duplicated gene couples since they can be easily generated using the SSD and WGD couples, and the list of genes contained in the network of choice that are present in the GitHub repository.

The manuscript is well-organized and is easy to follow. Figures are clear easy to understand.

We made a strong effort to make our manuscript and figures clear and understandable, and we are very pleased to hear that we reached our goal.

ANSWERS TO REVIEWER #2

The paper of Mottes et al. describes the long-term effects of two rounds of Whole Genome Duplication (WGD) at the dawn of vertebrate evolution on the architecture of the human gene regulatory networks. The authors integrated information on transcriptional regulation, miRNA regulation, and protein-protein interactions to analyse the role of both small and large-scale (whole genome) duplications on the structural properties of biological and regulatory networks. The authors conclude that both ancient WGD events played a substantial role in increasing the overall complexity of the vertebrate regulatory network by enhancing its combinatorial organization, with potential consequences on overall robustness and increase in biological complexity. I have read this paper with great interest. I think the paper is well written and, in my opinion, does a great job in introducing and explaining the aims of the study.

I have gone over the paper twice and could not come up with any major objections. As far as I can judge, the methodology and approach used warrants the conclusions and the authors have been careful not to overinterpret the results. All data are available and have been well described.

We are grateful to the Reviewer for the positive remarks.

Nevertheless, I do have the following comments.

On lines 521-522, the authors state that: "Most of the results mentioned above on duplication mechanisms are based on observations and experiments performed in simple model organisms like S. cerevisiae and A. thaliana. The new data on vertebrate WGD genes give us the unique opportunity to extend previous studies in a more complex setting." I believe the authors are wrong in assuming that A. thaliana is a simple(r) model organism. A. thaliana has more genes than human (I admit this does not say or mean much), but more importantly, A. thaliana has undergone and survived several whole genome duplications, probably more than any other organism, including human. So, it is not clear to this reviewer why the authors refer to A. thaliana as a simple(r) model organism? Also, in this respect, I have the feeling the authors should perhaps pay more attention to the (recent) literature on whole genome duplications in plants. Apart from these two ancient WGDs in vertebrates, and an additional one, about 300 mya, in the fish lineage, the great majority of genome duplications have been described for plants. For instance, although the authors do mention Arabidopsis a few times (but without going into detail, and often together with the, indeed, simpler yeast), in general I have the feeling that the authors neglect a little too much what is known about plants and WGDs. For instance, the authors write, on lines 455-457: "In this case, dosage balance (and thus duplicate retention) is granted by a substantial decrease in gene expression of the duplicated pair, which allows to re-balance gene dosage after duplication. Examples of this behaviour have been found both in yeast and in mammals.". There are several recent papers discussing dosage, gene balance, epigenetic remodeling, subgenome dominance etc. in plants as well, and probably (much) more than what has been described for animals and even yeast.

We thank the Reviewer for pointing out this issue. After a more thoughtful consideration of the matter, we agree on the fact that considering *A. thaliana* and plants in general as simpler might have been, indeed, too simplistic. We rephrased that section

and corrected this oversight. We also added some references in the revised manuscript, listed in the next section as [6–11], to address the concerns expressed by the Reviewer about the lack of a more extensive survey of related results in plants. The literature seems to confirm the general picture suggested in our work and by works on vertebrates and yeast in general, in particular concerning the role of dosage balance in gene retention after copy events and the role of WGDs in promoting (morphological) complexity.

We are well aware of the central importance of WGD events in the evolutionary history of plants, but we feel that a more detailed survey on the matter would be out of the scope of this work and might even confuse some readers. We tried to give a broad picture of WGD consequences in general for the sake of clarity, but we also tried to keep the discussion focused on vertebrates and humans in particular.

The authors conclude that "... and it is by now widely accepted that two rounds of whole genome duplication happened at the origin of the vertebrate lineage [1]. How these two global-scale events affected the gene regulatory networks is, however, still to be fully understood. Thanks to the recently published lists of WGD pairs [14, 16, 17], we had the possibility to tackle this problem. This paper quantifies the effects of WGD and SSD events on the structure of regulatory networks in human, and the results support the idea that these networks were significantly shaped by the two rounds of WGD at the beginning of the vertebrate lineage. Our analysis of network motifs specifically indicates that the two rounds of WGD contributed substantially to the overall regulatory redundancy, promoted synergy between different regulatory layers, and typically generated motifs that can be associated with complex functions." While I agree with this overall statement of the authors, I was hoping to see some more speculation on how this 'overall regulatory redundancy, synergy between different regulatory layers, and generated motifs associated with complex functions' might have 'helped' or facilitated the evolution of vertebrates in particular. Can these WGDs therefore be indeed linked to an increased complexity of vertebrates which likely would not have been possible without these WGDs, as has been suggested by Ohno (1970), or alternatively, could these WGDs and their effects on gene regulatory networks have reduced the risk of extinction as suggested by Crow and Wagner (2006), for instance. Although I understand this is not self-evident, a little more speculation on the possible biological or evolutionary consequences of the specific observations made in this study would be nice, in my opinion. The authors mention some genes (see for instance Fig. 8), part of some pathways, but a deeper discussion on for example how some specific gene(s) and their recruitment in a specific duplicated motif or pathway could have been important for vertebrate adaptation of evolution would be very interesting.

We thank the Reviewer for this thought-provoking remark. In order to better illustrate what the role of WGD events might have been in the evolution of complex traits in vertebrates, we added in the revised manuscript a more in-depth discussion of the evolution and significance of the RAR/RXR pathway. In our opinion it is a perfect example of the beneficial effects that WGD events introduced in the vertebrate regulatory networks and of their role in promoting complexity (morphological, in this specific case). The choice of this particular pathway was also driven by the extremely

high statistical significance of the overlap in regulatory interactions among the genes that constitute it. An hypergeometric test conducted on the common protein-protein interactions between any two genes in the RAR family gives a p-value of the order of $10e-35$ or less. To better ground our discussion of this example we also added some references, reported below as [1–5].

We thank the Reviewer for pointing us to the paper by Crow and Wagner [12], which indeed presents a very interesting analysis and was missing in our references. Our opinion, though, is that this work is not necessarily in full contrast with the work of Ohno and others. It might well be the case that WGD events help reduce the risk of extinction in the immediate, and only in a second moment and under specific circumstances actually act as a driver of complexity. This seems to be also the thesis of Crow and Wagner. We agree on the fact that the production of evolutionary novelty might not be the primary advantage conferred by WGD event right after they are retained. This should not affect, however, the final message of our work. WGD events seem to promote, in the long run, the retention of some gene regulatory configurations in a way which substantially differs, in many cases, from SSD events alone. We added the reference to this very interesting work in our Introduction, but we feel that more lengthy speculations on this matter could confuse some readers on the aim and results of our study. We would prefer to leave it as a hint for further research on the topic to the more interested readers.

The authors discuss motif enrichment in simple and duplicated networks, but I was wondering whether, for instance in duplicated networks, they have also considered underrepresentation of certain motifs? One could imagine that certain motifs, when duplicated, could be detrimental or would lead to maladaptation or lower fitness. Is this something the authors looked at or considered?

This is certainly a most relevant observation, we thank the Reviewer for bringing up this issue and allowing us to discuss it more in detail. The short answer would simply be that we did not find any strong under-representation for the duplicated motifs that we considered, but it is worth discussing this matter more in detail. We propose in the next paragraph a line of reasoning that, we hope, gives an intuitive justification of why we should expect to observe this situation.

The first thing to notice is that gene copy (both by SSD and WGD) naturally creates a lot of redundancy in regulations, and thus a lot of motifs. If a duplication is a beneficial one, we expect that a lot of the created redundancy will be retained. As a consequence, we will see a lot of motifs involving the duplicated couple, resulting in an over-representation of duplicated motifs. If the duplication is detrimental for the organism, instead, we expect that the duplicated gene will not be retained and we will only see one copy of that gene in the current network. This obviously means that we will not see that gene as a duplicate of another one, an thus it will not affect in any way the count of duplicated motifs. In the end, the only situation in which we could see an under-representation of duplicated motifs is the one where many

duplications are overall beneficial and thus retained, but common interactions between the duplicated gene couples are detrimental and hence strongly selected against by evolutionary forces. Although certainly not impossible, this situation would be very surprising at the least. Therefore we should expect to find hardly any duplicated motifs which are strongly depleted, in agreement with what we observe.

REFERENCES ADDED IN THE REVISED MANUSCRIPT

[1] Karen Niederreither and Pascal Dolle. Retinoic acid in development: towards an integrated view. *Nat Rev Genet*, 9(7):541–553, 2008.

[2] Juliana Gutierrez-Mazariegos, Eswar Kumar Nadendla, Daniela Lima, Keely Pierzchalski, Jace W. Jones, Maureen Kane, Jun-Ichi Nishikawa, Youhei Hiromori, Tsuyoshi Nakanishi, Miguel M. Santos, L. Filipe C. Castro, William Bourguet, Michael Schubert, and Vincent Laudet. A Mollusk Retinoic Acid Receptor (RAR) Ortholog Sheds Light on the Evolution of Ligand Binding. *Endocrinology*, 155(11):4275–4286, 2014.

[3] Mette Handberg-Thorsager, Juliana Gutierrez-Mazariegos, Stefan T. Arold, Eswar Kumar Nadendla, Paola Y. Bertucci, Pierre Germain, Pavel Tomancak, Keely Pierzchalski, Jace W. Jones, Ricard Albalat, Maureen A. Kane, William Bourguet, Vincent Laudet, Detlev Arendt, and Michael Schubert. The ancestral retinoic acid receptor was a low-affinity sensor triggering neuronal differentiation. *Sci Adv*, 4(2), 2018.

[4] Hector Escriva, Stephanie Bertrand, Pierre Germain, Marc Robinson-Rechavi, Muriel Umbhauer, Jerome Cartry, Marilyne Duffraisse, Linda Holland, Hinrich Gronemeyer, and Vincent Laudet. Neofunctionalization in vertebrates: The example of retinoic acid receptors. *PLoS Genetics*, 2(7):955–965, 2006.

[5] Yasuo Sakai, Chikara Meno, Hideta Fujii, Jinsuke Nishino, Hidetaka Shiratori, Yukio Saijoh, Janet Rossant, and Hiroshi Hamada. The retinoic acid-inactivating enzyme cyp26 is essential for establishing an uneven distribution of retinoic acid along the anterio-posterior axis within the mouse embryo. *Genes Dev*, 15(2):213–225, 2001.

[6] Michael J. Song, Barney I. Potter, Jeff J. Doyle, and Jeremy E. Coate. Gene Balance Predicts Transcriptional Responses Immediately Following Ploidy Change in Arabidopsis thaliana. *Plant Cell*, 32(5):1434–1448, 2020.

[7] Lex E. Flagel and Jonathan F. Wendel. Gene duplication and evolutionary novelty in plants. *New Phytol*, 183(3):557–564, 2009.

[8] Feng Cheng, Jian Wu, Xu Cai, Jianli Liang, Michael Freeling, and Xiaowu Wang. Gene retention, fractionation and subgenome differences in polyploid plants. *Nat Plants*, 4(5):258–268, 2018.

[9] Hui Guo, Tae-Ho Lee, Xiyin Wang, and Andrew H. Paterson. Function Relaxation Followed by Diversifying Selection after Whole-Genome Duplication in Flowering Plants . *Plant Physiol*, 162(2):769–778, 2013.

[10] James Birchler and Reiner Veitia. Protein–protein and protein–dna dosage balance and differential paralog transcription factor retention in polyploids. *Front Plant Sci*, 2:64, 2011.

[11] Michaël Bekaert, Patrick P. Edger, J. Chris Pires, and Gavin C. Conant. Two-Phase Resolution of Polyploidy in the Arabidopsis Metabolic Network Gives Rise to Relative and Absolute Dosage Constraints. *Plant Cell*, 23(5):1719–1728, 2011.

[12] Karen D. Crow and Günter P. Wagner. What Is the Role of Genome Duplication in the Evolution of Complexity and Diversity? *Molecular Biology and Evolution*, 23(5):887–892, 2005.
